# An *in silico *model of the ubiquitin-proteasome system that incorporates normal homeostasis and age-related decline

**DOI:** 10.1186/1752-0509-1-17

**Published:** 2007-03-21

**Authors:** Carole J Proctor, Maria Tsirigotis, Douglas A Gray

**Affiliations:** 1 School of Clinical and Medical Sciences-Gerontology, Newcastle University, UK; 2 Centre for Integrated Systems Biology of Ageing and Nutrition, Newcastle University, UK; 3 Ottawa Health Research Institute, Ottawa, Canada; 4 Department of Biochemistry, Microbiology and Immunology, University of Ottawa, Ottawa, Canada

## Abstract

**Background:**

The ubiquitin-proteasome system is responsible for homeostatic degradation of intact protein substrates as well as the elimination of damaged or misfolded proteins that might otherwise aggregate. During ageing there is a decline in proteasome activity and an increase in aggregated proteins. Many neurodegenerative diseases are characterised by the presence of distinctive ubiquitin-positive inclusion bodies in affected regions of the brain. These inclusions consist of insoluble, unfolded, ubiquitinated polypeptides that fail to be targeted and degraded by the proteasome. We are using a systems biology approach to try and determine the primary event in the decline in proteolytic capacity with age and whether there is in fact a vicious cycle of inhibition, with accumulating aggregates further inhibiting proteolysis, prompting accumulation of aggregates and so on. A stochastic model of the ubiquitin-proteasome system has been developed using the Systems Biology Mark-up Language (SBML). Simulations are carried out on the BASIS (Biology of Ageing e-Science Integration and Simulation) system and the model output is compared to experimental data wherein levels of ubiquitin and ubiquitinated substrates are monitored in cultured cells under various conditions. The model can be used to predict the effects of different experimental procedures such as inhibition of the proteasome or shutting down the enzyme cascade responsible for ubiquitin conjugation.

**Results:**

The model output shows good agreement with experimental data under a number of different conditions. However, our model predicts that monomeric ubiquitin pools are always depleted under conditions of proteasome inhibition, whereas experimental data show that monomeric pools were depleted in IMR-90 cells but not in ts20 cells, suggesting that cell lines vary in their ability to replenish ubiquitin pools and there is the need to incorporate ubiquitin turnover into the model. Sensitivity analysis of the model revealed which parameters have an important effect on protein turnover and aggregation kinetics.

**Conclusion:**

We have developed a model of the ubiquitin-proteasome system using an iterative approach of model building and validation against experimental data. Using SBML to encode the model ensures that it can be easily modified and extended as more data become available. Important aspects to be included in subsequent models are details of ubiquitin turnover, models of autophagy, the inclusion of a pool of short-lived proteins and further details of the aggregation process.

## Background

Ageing is due to the gradual accumulation of unrepaired random molecular faults, which leads to an increased fraction of damaged cells and eventually to the functional impairment of older tissues and organs [reviewed in [1]]. Cellular components are continuously renewed by the processes of catabolism and resynthesis as they wear out or become damaged. However the mechanisms of renewal are not perfectly efficient and over time there may be an increase in damaged molecular structures. For example, there is an accumulation of incompletely degraded intralysosomal waste material, known as lipofuscin, in postmitotic cells [2]. In 2001, Terman proposed the "garbage catastrophe theory" of ageing [3] which states that the process of ageing may derive from imperfect clearance of oxidatively damaged, relatively indigestible material, the accumulation of which further hinders cellular catabolic and anabolic functions. Terman points out that this theory applies mainly to postmitotic cells and not to proliferating cells. This is supported by the evidence that tissues composed of postmitotic cells such as the heart and brain show the most pronounced age-related changes [4,5], whereas tissues with constantly proliferating cell populations such as the intestinal epithelium show only minor changes with age [6]. A possible reason for this difference is that during cell growth and division, most cellular structures are renewed and there is a dilution of any undigested material.

Terman mainly applied his theory to the lysosomal pathway of degradation. He suggested that lysosomes gradually accumulate lipofuscin and that the lipofuscin granules attract lysosomal enzymes which, however, fail to degrade the pigment. This leads to a shortage in lysosomal enzymes, resulting in a decrease in degradation by this pathway. However, the major pathway for the rapid degradation of proteins is the ubiquitin-proteasome pathway. Proteins need to be degraded either because they are short-lived e.g. key regulatory proteins or because they have become damaged and partially unfolded. Partially unfolded proteins have exposed hydrophobic surfaces and are liable to form aggregates. Molecular chaperones bind to the exposed surfaces and an attempt is made to refold the protein. If refolding is unsuccessful then chaperones assist in the removal of these proteins and so prevent their aggregation [7].

Interestingly, proteasomes themselves are degraded by the lysosomal system and there is evidence for cross-talk between the two degradation pathways [8,9]. It has been observed that there is a progressive decline in the overall proteolytic capacity of the cell with age [10,11] resulting in an accumulation of oxidized and cross-linked proteins [12,13]. These observations have been linked to an increase in free radicals with age [11]. However, there is also evidence that free radicals do not increase with age [14,15], suggesting that the observed increase in damaged proteins may be a result of a decline in removal of damage rather than an increase in the rate of damage itself. Proteins that have been damaged by free radicals appear resistant to proteolytic degradation and can act as inhibitors of lysosomal hydrolases and proteasome activity [16]. The small aggregates which precede the formation of mature amyloid fibres are particularly pathogenic in many amyloid diseases [17]. Sitte et al[12] found a dramatic decline of proteasome activity, but not of proteasome enzyme levels in nondividing human BJ fibroblasts during hyperoxic ageing. Such a decline is not a cell culture artefact because it has been observed in many tissues including cardiac and skeletal muscle, skin, lens and brain (reviewed in [18]). Sitte et al[12] did not observe a decrease in lysosomal activity with time in nondividing BJ fibroblasts. However, both proteasome and lysosomal activity decreased in proliferating BJ fibroblasts [13].

It has been reported that aggregated protein directly impairs the function of the ubiquitin-proteasome system [19,20]. Another set of data indicates that aggregation occurs when the capacity of the proteasome degradation pathway is exceeded but no distinction could be drawn between the possibility of increased substrate expression or a decline in proteasome activity [21]. Therefore, it is not clear whether aggregated protein directly impairs proteolysis or whether its presence is a symptom of impaired proteolysis. The issues of cause versus effect require further investigation particularly in the role of ageing.

Many neurodegenerative diseases are characterised by the presence of distinctive ubiquitin-positive inclusion bodies in affected regions of the brain. These inclusions consist of insoluble, unfolded, ubiquitinated polypeptides that fail to be targeted and degraded by the proteasome [22]. It has been suggested that cells possess a protective mechanism to sequester and isolate toxic misfolded cytoplasmic intermediates by delivering them to ubiquitin-rich structures, called aggresomes at the microtubule-organising centre (MTOC) [21]. Nuclear inclusions may serve a similar function, sequestering aggregation-prone polypeptides into relatively inert higher-order complexes. It has been demonstrated that misfolded proteins are less toxic to cells capable of forming nuclear inclusions [23].

In order to be recognised and degraded by the 26S proteasome, proteins must first be tagged with chains of four or more ubiquitin molecules. Ubiquitin is a small protein found in all eukaryotic organisms. Ubiquitination occurs as a result of the sequential activity of three classes of enzymes, E1 (ubiquitin activating enzyme), E2 (ubiquitin conjugating enzyme), and E3 (ubiquitin protein ligase). In the first step, ubiquitin is activated through ATP hydrolysis mediated by an E1 enzyme. The activated ubiquitin is then transferred to an E2 (ubiquitin conjugate) enzyme. There are approximately 50 different E2 proteins in mammalian cells [24] that have limited substrate specificities. E2 bound to ubiquitin then forms a complex with an E3 (ubiquitin ligase) enzyme. There are hundreds of E3 proteins [24] specific for different substrates. Three classes of E3 proteins have been identified: the HECT (homologous to E6-AP C-terminus), the RING (really interesting new gene) finger, and the U-box domain types (see review by Robinson and Ardley [25]). The following describes the action of a HECT E3. As well as binding to the E2-ubiquitin complex, E3 binds to the protein which is to be degraded. The activated ubiquitin is then transferred to the substrate with the release of E2 and E3. At this stage the protein is mono-ubiquitinated. Further ubiquitin is then attached to form a chain by the further action of E1 and E2 enzymes. A ubiquitin chain of at least four ubiquitin molecules has physical affinity for the proteasome and delivers the substrate for degradation in an ATP-dependent manner [26]. During degradation, the ubiquitin is released for recycling and the protein is cleaved into short peptides, which are then reduced to amino-acids by cytosolic proteases. Note that for simplicity our model ignores alternative mechanisms of chain assembly (conjugation of preassembled chains onto substrates etc., as discussed in [27], and does not require the activity of E4 enzymes, which for some substrates may promote chain elongation [28].

A large number of de-ubiquitinating enzymes (DUBs) are also found within all eukaryotic cells [29]. In addition to processing the primary pro-protein translation products of the ubiquitin genes DUBs may edit polyubiquitin chains on substrates and thereby prolong the half-life of such substrates by preventing the formation of threshold length chains required for proteasome binding.

A model of protein turnover and the role of the molecular chaperone Hsp90 in maintaining protein homeostasis has previously been developed [30]. This model contains a pool of proteins in its correctly folded native state (represented by a species NatP). At any point in time, a native protein can become misfolded with the rate of this reaction depending on the level of reactive oxygen species (ROS) within the cell (higher levels of ROS lead to an increase in the rate of misfolding). The pool of misfolded proteins is represented by a species MisP. There are three possible outcomes for a misfolded protein. The first possibility is that a misfolded protein binds to Hsp90 and once bound, it can either be refolded and return to the pool of native protein or it may dissociate from Hsp90 and so remain in its misfolded state. Secondly, a misfolded protein may be degraded and so removed. Thirdly, a misfolded protein may bind to another misfolded protein to form a small aggregate (or bind to a previously formed aggregate). The model contains details of the regulation of Hsp90 through its interaction with Heat Shock Factor-1 (HSF1). However, no detail is included of either the degradation pathway or the process of aggregation.

**Figure 1:**
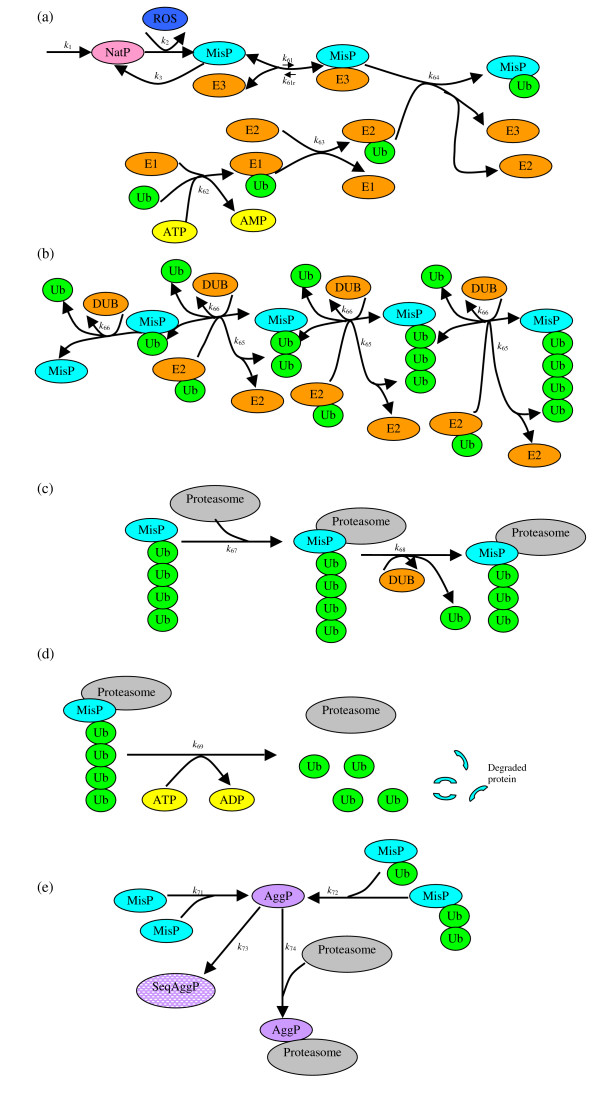
**Network diagram of the ubiquitin-proteasome model**. (a) Reactions leading to the first ubiquitination step; (b) reactions of chain lengthening and shortening (only shown for chains up to length four); (c) binding of substrate with polyubiquitin chains (of length four or more) to proteasome and chain shortening of bound substrates by DUBs which leads to release from proteasome when chain is less than four; (d) degradation of substrates by proteasome in ATP-dependent manner releasing ubiquitin for recycling; (e) formation of aggregated proteins, sequestering of aggregates and proteasome inhibition by aggregates.

The aim of this paper is to show that the model previously used to simulate chaperone function can be extended to model ubiquitin-mediated proteolysis, incorporating molecular details of this degradation system. We have also sought to incorporate details of the aggregation process. One approach would be to take the computer code for the chaperone model and then extend the code. However, a better approach is to build a new and separate model that can be tested individually and then linked with the chaperone model. A network diagram of the ubiquitin-proteasome model is given in Figure 1. Details of how the model was built are given in the Methods section.

## Results

### Normal conditions

**Figure 2:**
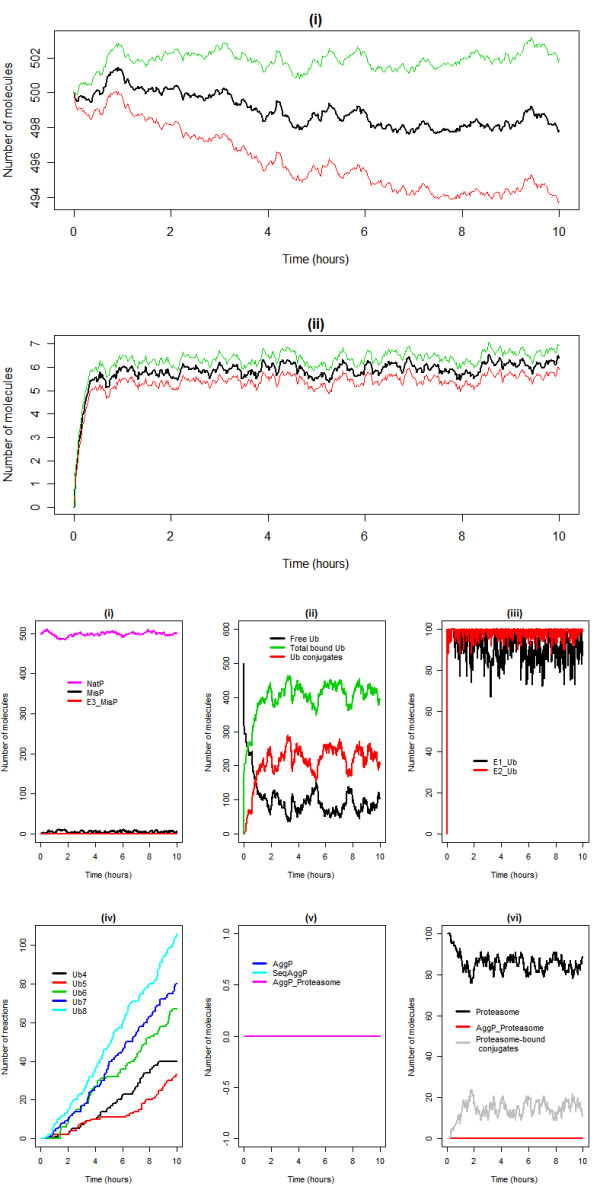
**Model output under normal conditions**. (a) Stochastic variation. Simulations were repeated 100 times and the mean for two of the species plotted. The black lines show the mean values, the green and red lines show the upper and lower 95% confidence interval respectively. (i) Native protein (NatP). (ii) Misfolded protein (MisP). (b) Results are shown for a typical simulation, for a 10 hour period. (i) Level of native protein (NatP), misfolded protein (MisP), and misfolded protein bound to E3 (E3_MisP). (ii) Ubiquitin pools. (iii) Ubiquitin bound to E1 and E2 (E1_Ub and E2_Ub respectively). (iv) Cumulative number of degradation reactions for different length of ubiquitin chains bound to substrate. (v) Accumulation of aggregated protein (AggP), sequestered aggregated protein (SeqAggP) and aggregated protein bound to proteasomes (AggP_Proteasome). (vi) Available pool of proteasomes, number of proteasomes bound by substrates and number of proteasomes bound by aggregated proteins.

The model was simulated with the initial values and parameter values given in Tables 1 and 2 respectively. Since the model is stochastic, each simulation produces slightly different results even when starting with the same set of initial values and parameter values. Figure 2a shows the random fluctuations for two of the model species when simulations are repeated 100 times. The model predicts that the level of native protein remains fairly constant, with a mean value of 499.0 with some small fluctuations due to stochastic effects and the level of misfolded protein remains low with a mean of 5.8 misfolded proteins at any one time. The mean values were calculated over a five hour time period from 100 simulations. The model output for a range of species from a single simulation is shown in Figure 2b. Initially there is a very steep decline in monomeric ubiquitin corresponding to the pool of ubiquitin that binds to E1 and E2. The pool of free ubiquitin further declines as misfolded protein is ubiquitinated. When the system settles down after about two hours, the pool of ubiquitin conjugates is larger than the pool of monomeric ubiquitin which agrees with experimental data (Fig 2b(ii)). The fluctuations in the ubiquitin pools are mainly due to the fluctuations in the amount of ubiquitin bound to misfolded proteins including those bound at the proteasome due to the opposing reactions of ubiquitination and de-ubiquitination. Some of the variation is also caused by fluctuations in the amount of E1 bound to ubiquitin (see Fig 2b(iii)). We kept track of the number of degradation reactions and the length of ubiquitin chain at the time of degradation. We found that the number of degradation reactions increases with the length of chain (Fig 2b(iv)). Since the degradation rate does not depend on chain length, then the reason that misfolded proteins with longer polyubiquitin chains are more likely to be degraded is that they spend more time physically associated with the proteasome. Under normal conditions, there is no formation of aggregates and so no inhibition of the proteasome (Fig 2b(v),(vi)). The levels of available unbound proteasomes fluctuate between 75 and 100, as a result of varying levels in bound polyubiquitinated protein.

We carried out a sensitivity analysis to see how changing each of the model parameters affects the simulation output. The only parameters which have a significant effect on the results when increased or decreased by an order of magnitude were *k*_1_, *k*_2_, *k*_67_, *k*_68_, and *k*_69_. Varying the parameter for protein synthesis, *k*_1_, affects the levels of native protein but only an increase in *k*_1 _has any affect on any of the other species. Increasing protein synthesis leads to a depletion in monomeric ubiquitin pools due to the increase pool of protein requiring degradation. An increase in the parameter for misfolding, *k*_2_, leads to an increase in misfolded protein, a decrease in native protein, all the ubiquitin ending up in conjugates and an increase in degradation. Conversely, a decrease in *k*_2_, leads to higher levels of native protein, more monomeric ubiquitin, less ubiquitin conjugates and less degradation. Increasing the parameter for binding of polyubiquitinated misfolded protein to the proteasome, *k*_67_, has no effect on the model output, but a decrease in *k*_67 _leads to a depletion in monomeric ubiquitin pools, an increase in unbound polyubiquitinated misfolded protein and less degradation. Increasing the parameter for the chain shortening of polyubiquitinated misfolded protein, *k*_68_, has the same effect as decreasing *k*_67_. However, decreasing *k*_68 _has little effect on model output except that nearly all the degradation reactions are via the substrates with the longest ubiquitin chain. Increasing the parameter for proteasome activity, *k*_69_, leads to more degradation and less ubiquitin conjugates, whereas decreasing *k*_69 _has the opposite effect and also results in depletion of monomeric ubiquitin due to less recycling of ubiquitin.

### Proteasome inhibition

**Figure 3:**
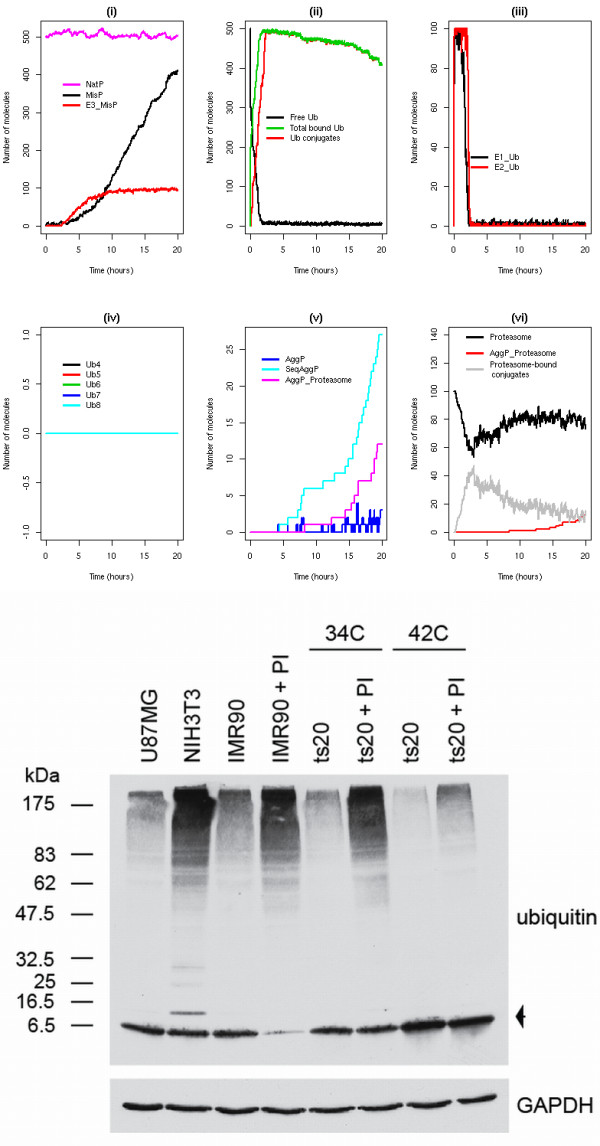
**Inhibiting proteasome activity**. Results are shown for a typical simulation, for a 20 hour period (a) **Model output **(*k*_69 _= 0, all other parameters as in Tables 2 and 3). (i) Level of native protein (NatP), misfolded protein (MisP), and misfolded protein bound to E3 (E3_MisP). (ii) Ubiquitin pools. (iii) Ubiquitin bound to E1 and E2 (E1_Ub and E2_Ub respectively). (iv) Cumulative number of degradation reactions for different length of ubiquitin chains bound to substrate. (v) Accumulation of aggregated protein (AggP), sequestered aggregated protein (SeqAggP) and aggregated protein bound to proteasomes (AggP_Proteasome). (vi) Available pool of proteasomes, number of proteasomes bound by substrates and number of proteasomes bound by aggregated proteins. (b) **Survey of ubiquitin pools in various cell lines**. Western blot analysis of cell extracts from U87MG, NIH-3T3, IMR90 and ts20 cells with an antibody raised against ubiquitin. The pool of conjugated ubiquitin (vertical line) was found to differ between cell lines with the highest levels detected in NIH-3T3 cells. Comparable levels of conjugated ubiquitin were detected in IMR90, U87MG and ts20 cells cultured at 34°C. ts20 cells cultured at 42°C for 3 hours had the least detectable levels of poly-ubiquitinated conjugates. Whereas a shift from monomeric (arrowhead) to conjugated ubiquitin (vertical line) was observed in IMR90 cells treated with proteasome inhibitor for 3 hours, no difference in the pool of monomeric ubiquitin was observed in ts20 cells treated with the inhibitor. The membrane was re-probed with an antibody directed against GAPDH which served as a loading control (boxed panel).

We carried out an *in silico *experiment of inhibiting the proteasome by setting the parameter *k*_69 _= 0. The model predicts that there is initially a build up of ubiquitinated protein and that the pool of monomeric ubiquitin is depleted. The depletion of ubiquitin is followed by a steady rise in the level of misfolded protein (Fig 3a). Note that our experimental data invariably show a decline in monomeric ubiquitin in IMR90 cells but that the results are more variable in ts20 cells; in some experiments there is no evidence of depletion in monomeric ubiquitin in the ts20 cells (Fig 3b) This suggests that there is compensatory upregulation of ubiquitin in ts20 cells after proteasome inhibition. Currently our model assumes that the total pool of ubiquitin remains constant but the model can easily be extended to include detail of ubiquitin turnover (Proctor, Tsirigotis, & Gray, in preparation). The model predicts that the level of native protein is not affected, as protein synthesis continues at the same rate and so replaces any protein which becomes misfolded (Fig 3a). Aggregated protein starts to form after five hours and there is a low level of aggregates bound to proteasomes.

We also varied each of the parameters in turn to see which parameters affect the kinetics of aggregation. We found that apart from the parameters actually involved in the aggregation steps (*k*_71_, *k*_72_, *k*_73_, *k*_74_), the only parameters which affect aggregation are *k*_1_, *k*_2_, *k*_3 _and *k*_61_, Either an increase in protein synthesis (*k*_1_) or in the misfolding rate (*k*_2_) leads to an increase in aggregated protein which in turn leads to both an increase in sequestered aggregated protein (SeqAggP) and binding of aggregated protein to the proteasome (AggP_Proteasome). This is due to the increase level of misfolded protein which can not be degraded. On the other hand an increase in refolding (*k*_3_) leads to a reduction in aggregation. Increased refolding could occur if there was an upregulation in molecular chaperones. Increasing the rate of binding of E3 to misfolded protein (*k*_61_) also reduces the level of aggregation suggesting that, if a particular protein was involved in aggregation, then overexpressing the relevant E3 for a misfolded protein could help to prevent the accumulation of aggregates. However, aggregates are usually composed of a variety of proteins, so this approach may not be feasible. As expected, increasing the rate of aggregation by either increasing *k*_71 _or *k*_72 _leads to more aggregation, and varying the parameters *k*_73 _or *k*_74 _changes the ratio of SeqAggP to AggP_Proteasome.

### Parameters which affect protein turnover

**Figure 4:**
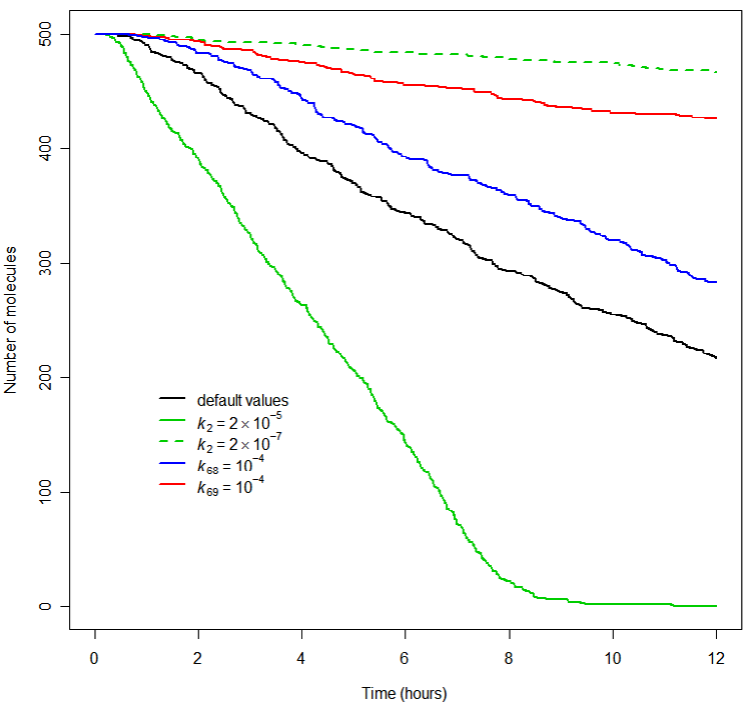
**Model parameters that affect protein half-life**. Results are shown for a typical simulation, for a 12 hour period. Protein synthesis was blocked by setting *k*_1 _= 0 with all other parameters as in Tables 2 and 3 except where indicated. The curves show the decline in total protein with time. The half-life is the time when the total protein level is equal to 250.

By setting the rate of protein synthesis, *k*_1 _to zero, we can see which parameters affect the protein half-life. Figure 4 shows that with the default parameters, the protein half-life in this model is about 10 hours. We both increased and decreased each of the model parameters in turn by an order of magnitude. We found that only three parameters have an effect on protein half-life: *k*_2_, *k*_68 _and *k*_69_. The parameter for the protein misfolding reaction (*k*_2_) is most sensitive with a ten-fold increase leading to a ten-fold decrease in protein half-life, and a ten-fold decrease leading to a ten-fold increase in half-life (Fig 4 green lines). The parameter for proteasome activity (*k*_69_) only has an effect when decreased, with a ten-fold decrease leading to a four-fold increase in protein half-life (Fig 4, red line). The reason that increasing *k*_69 _has no effect is that we chose parameters so that under normal conditions proteasomes are working well below their maximum capacity. A ten-fold increase in the rate of shortening the chains of polyubiquitinated misfolded protein bound to the proteasome (*k*_68_), leads to a slight increase (× 1.4) in protein half-life (Fig 4, blue line). This is due to more bound conjugates being released instead of being degraded by the proteasome.

### Shutting down E1 activity

**Figure 5:**
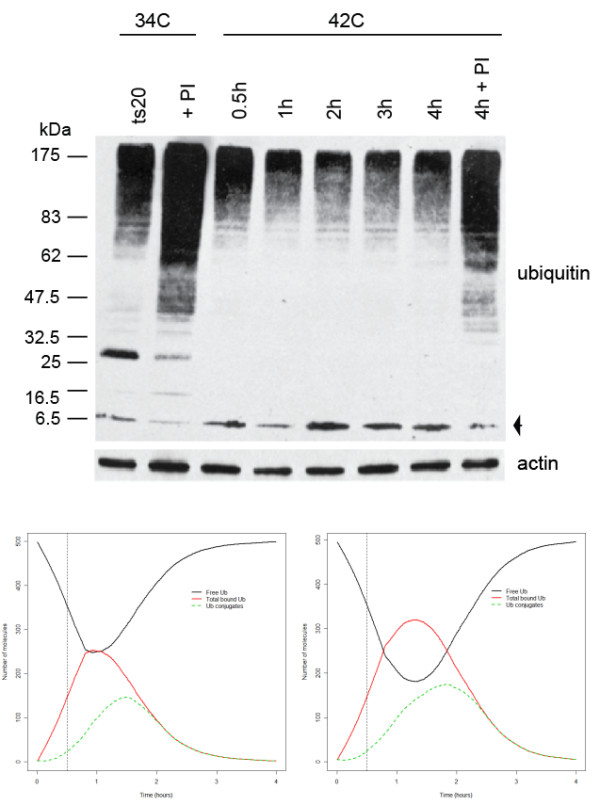
**Shutting down E1 activity in ts20 cells**. (a) **Time course of conversion of conjugates into monomeric ubiquitin pool **Western blot analysis of cell extracts from ts20 cells cultured either at 34°C or 42°C for the indicated time with an antibody recognizing ubiquitin. The analysis revealed a detectable conversion of ubiquitin conjugates into monomeric ubiquitin in cells cultured at 42°C for 2 hours. Maintaining the cells at 42°C for an additional 1 or 2 hours did not significantly enhance the conversion of conjugates into the monomeric pool. The addition of proteasome inhibitor in the culture medium of cells cultured at 42°C blocked the conversion of poly-ubiquitinated conjugates and resulted in their enhanced detection. The membrane was re-probed with an actin specific antibody which served as a loading control (boxed panel). (b) **Model output **E1 activity was blocked at time = 0.5 hours by setting the parameter *k*_62 _= 0 at this time point (indicated by dashed vertical line on the graph). All other parameters as in Tables 2 and 3. The simulations were repeated 100 times and the curves show the mean values. Ub conjugates includes all ubiquitinated misfolded protein both free and bound to the proteasome; total bound Ub includes ubiquitin bound to E1 and E2 enzymes in addition to ubiquitinated misfolded proteins. (c) As (b) except ten-fold lower DUB activity on polyubiquitinated proteasome-bound substrates (*k*_68 _= 1.0 × 10^-6^)

The ts20 cell line is a Chinese hamster ovary line in which a temperature-sensitive mutation in E1 allows functional inactivation of this enzyme at the nonpermissive temperature [31]. Figure 5 shows the results of the wet-lab and *in silico *experiments wherein E1 activity is eliminated, and subsequent conjugation of ubiquitin precluded. When ts20 cells are cultured at 34°C and then subject to a temperature shift to 42°C, there is initially no decline in ubiquitin conjugates (Figure 5a, 30 minutes). Then after 1 to 2 hours, the conjugates start to disappear and there is a corresponding increase in the monomeric pool of ubiquitin. The model predicts that when E1 activity is stopped (at time = 0.5 hours), ubiquitin conjugates continue to increase for a further half hour and then they start to decline, with levels becoming low after 2 hours (from cessation of E1 activity) and disappearing completely after 3 hours as shown in Fig 5b. Lowering the rate of DUB activity ten-fold, slowed down the removal of ubiquitin conjugates, so that it took 2.5 hours, after cessation of E1 activity for the conjugates levels to reach low levels and 3.5 hours to completely disappear (Fig 5c)

## Discussion

We have built a computer model of the ubiquitin-proteasome system using an iterative process of model-building and validation of the model against experimental data. The initial conditions and parameters for the model were chosen so that the model output fitted the experimental data under normal conditions. The model was then validated by mimicking the experimental conditions such as proteasome inhibition and shutting down E1 activity. For example, with our original choice of parameters our model predicted that if E1 activity is shut down, then there is an almost immediate conversion of ubiquitin conjugates to monomeric ubiquitin. However, experimental data show that this conversion takes about two hours (Fig 5a). Therefore, we adjusted the parameters to reduce the rates of the reactions involved in ubiquitination, de-ubiquitination and degradation until the model output fitted the experimental data.

With this final set of parameters, the model output also agrees with experimental data under normal conditions and when the proteasome is inhibited. However, under conditions of proteasome inhibition our model only agrees with data for IMR-90 cells and not ts20 cells with respect to the pool of monomeric ubiquitin. IMR-90 cells are diploid fibroblast-like cells isolated from human fetal lung [32]; they are nontransformed and with prolonged culture will reach the Hayflick limit and undergo senescence. The ts20 cell line is an immortalized line derived from the E36 Chinese hamster ovary line [31]. Whereas cell lines were found to be variable with respect to ubiquitin content (Fig 3b) there did not seem to be a correlation between ubiquitin content and immortalization or oncogenic transformation (comparing the normal diploid IMR-90 to the human glioblastoma line U87MG, for example). It may be that the robust growth of transformed cells places demands on ubiquitin pools and that such cells have devised mechanisms to maintain or replenish their pools; if so the IMR-90 results may be a more accurate representation of normal ubiquitin homeostasis. In order to be able to use our model for different cell types (including transformed cells) it will be necessary to add detail of ubiquitin turnover instead of assuming a constant pool.

We carried out a sensitivity analysis to see which parameters affect the model output, not only under normal conditions but also under experimental conditions such as proteasome inhibition and shutting down E1 activity. Under normal conditions, pools of native protein were affected by changing the protein synthesis or misfolding rate, and pools of monomeric ubiquitin were affected by changing the rate of polyubiquitinated misfolded binding to the proteasome, the rate of chain shortening of polyubiquitinated misfolded protein bound to proteasomes, or the rate of proteasome activity. Under conditions of proteasome inhibition, increasing the rate of either protein synthesis or protein misfolding, or decreasing the rate of either refolding or binding of misfolded proteins to E3 led to an increase in protein aggregation (and vice versa). These results suggest that the availability of chaperones and E3 enzymes are important in maintaining protein homeostasis.

We also examined which parameters affect protein half-life. The most important parameter in this respect was the parameter for misfolding with a ten-fold increase/decrease leading to a ten-fold decrease/increase in protein half-life. Since the misfolding reaction can be driven by the level of ROS in the cell, our model would predict a similar outcome by changing ROS levels, with an increase in ROS leading to higher misfolding and an increase in degradation, consistent with current thought linking protein oxidation and ageing [33]. Our model predicts that a ten-fold decrease in the rate of proteasome activity leads to a four-fold increase in protein half-life but that an increase in proteasome activity did not decrease protein half-life. It has been suggested that since proteasome levels can only increase slowly due to their slow assembly time, that they must have residual capacity for degrading proteins when cells are stressed [34]. Our model allows for this residual capacity and explains why a ten-fold increase in proteasome activity has no effect on protein half-life.

There are also many other modifications that we could make to the model. For example, we have assumed that E3 binds to misfolded protein in one simple step; however it is likely that when a native protein misfolds, the first step would be for a molecular chaperone (e.g. Hsp90) to bind to the exposed hydrophobic surface to prevent inappropriate interactions. We could easily add this detail by including the following steps:

MisP + Hsp90 ↔ Hsp90_MisP (reversible reaction)

Hsp90_MisP + E3 → E3_MisP + Hsp90

Similarly, we could include the possibility that chaperones aid in delivery of ubiquitinated proteins to the proteasome.

Our model includes a HECT E3, but it would be easy to adapt the model for other types of E3 enzymes. For example if E3 is a RING ligase, then it has to be in complex with E2 before it can bind to the misfolded protein. This could be represented by the following reactions:

E3 + E2_Ub ↔ E2_E3_Ub (reversible reaction)

MisP + E2_E3_Ub → E2_E3_MisP_Ub

E2_E3_MisP_Ub → MisP_Ub + E2 + E3

Our model only includes one pool of long-lived proteins and so far ignores the fact that the majority of proteasome activity is involved in the turnover of short-lived regulatory proteins and also the elimination of incorrect newly-synthesised proteins. It would make the model more realistic to include an additional pool of short-lived proteins and this work is currently in progress.

We have so far restricted our models of protein turnover to the ubiquitin-proteasome system; however work is currently in progress to develop other models of protein turnover. It will be important to incorporate models of lysosomal pathways since studies suggest that protein aggregates such as mutant huntingtin are removed by autophagy [reviewed in [35]].

We have included the possibility of aggregated protein inhibiting proteasomes. Another possible cause of proteasome inhibition is via unaggregated damaged protein which could also sequester proteasomes. It would be fairly straight forward to add this extra detail to our current model. By running simulations over long time periods, the models can then be used to see how proteasome activity is affected by damaged and/or aggregated protein. This would be more realistic, than simply setting the parameter for proteasome activity to zero.

Our model predicted that levels of native protein did not change when misfolded protein levels rise, but in reality we would not expect total protein levels to continually rise. This prediction was due to our assumption of a constant protein synthesis rate. We could later include detail of feedback mechanisms such as the unfolded protein response which downregulates translation when unfolded protein accumulates in the endoplasmic reticulum.

We have encoded our model using SBML which not only allows for easy portability but also enables the model to be modified and extended in a very straight-forward way. The model is available on the BASIS website and can be freely accessed from the public repository, so that the interested reader can run simulations, make their own modifications, or download the SBML code. The BASIS website has a stochastic simulator and a database for storing models and simulation results.

Computer models of other molecular mechanisms involved in ageing are being developed at Newcastle University, for example the role of mitochondria, the antioxidant system, and DNA damage signalling pathways. Methods to link models in an automated way are also under development which will greatly facilitate the development of integrated models. For example, our model of the ubiquitin-proteasome system could be linked to a model of the mitochondria. This would be very valuable since neurodegeneration is not only associated with an increase in aggregated protein but also an increase in damaged mitochondrial DNA [36].

## Conclusion

We have developed a model of the ubiquitin-proteasome system using an iterative approach of model building and validation against experimental data. We have used SBML to encode the model to ensure that it can be easily modified and extended as more data become available. Important aspects to be included in subsequent models are details of ubiquitin turnover, models of autophagy, the inclusion of a pool of short-lived proteins and further details of the aggregation process. The model and its extensions will be an invaluable aid to further our understanding of the cellular mechanisms involved in maintaining protein homeostasis and how it is disturbed during ageing.

## Methods

### Experimental Procedures

#### Cell culture

U87MG human glioblastoma cells, NIH-3T3 mouse fibroblasts and IMR90 human fibroblasts were maintained in 5% CO_2 _at 37°C in Dulbecco's modified Eagle's medium (DMEM) supplemented with 10% of a 2:1 mixture of fetal bovine and donor bovine serum. ts20 cells harbouring a temperature sensitive E1 ubiquitin activating enzyme (a gift from Dr. Sergio Grinstein, University of Toronto) were maintained in 5% CO_2 _at 34°C in DMEM as above. Inactivation of E1 was achieved by incubating the cells at 42°C for the indicated time. When proteasome inhibitor (Calbiochem, San Diego, CA, USA) was used, it was added to the culture medium 3 or 4 hours prior to harvesting the cells for western analysis at a final concentration of 50 μM.

#### Preparation of cell extracts for western blot analysis

Cells were harvested in protein lysis buffer (20 mM Tris-HCl pH 7.5, 150 mM NaCl, 1% Nonidet P-40 (Sigma, St. Louis, Missouri, USA), 0.5 mM EDTA, 5% glycerol) containing the following protease and phosphates inhibitors: phenylmethylsulfonyl fluoride, leupeptin (Sigma, St. Louis, Missouri, USA), aprotinin (Sigma, St. Louis, Missouri, USA), sodium fluoride (NaF), sodium pyrophosphate (NaPPi) and N-ethylmaleimide. The cell extracts were sonicated and centrifuged at 14,000 rpm for 20 minutes at 4°C to pellet cellular debris. The soluble fractions were recovered and the protein concentration was determined using the Bradford protein assay (Bio-Rad Laboratories Inc., Mississauga, Ontario, Canada). 40 μg of cytoplasmic extracts were then resolved on a two-phase SDS-polyacrylamide gel (15 and 8%) and electroblotted onto a hybond C nitrocellulose membrane (Amersham Pharmacia Biotech, Baie D'Urfé, Québec, Canada). The membranes were autoclaved on a liquid cycle for 45 minutes to enhance the detection of poly-ubiquitinated proteins, stained with Ponceau S (Sigma, St. Louis, Missouri, USA) and analyzed by western blotting with the indicated antibody. The proteins were visualized by a horseradish peroxidase method using the ECL kit from Kirkegaard and Perry Laboratories Inc., Gaithersburg, Maryland, USA.

#### Antibodies

The rabbit polyclonal antibody used to detect ubiquitin was purchased from Dako. The mouse monoclonal antibodies recognizing actin and GAPDH were from Sigma (St. Louis, Missouri, USA) and Stressgen Bioreagents (Victoria, British Columbia) respectively.

### The Model

The model was built by first drawing a diagram of the system (see Figure 1) and listing all the elements of the model and the interactions between them. We then specified the initial amount of each element, the rate laws and the values of the parameters. With this information, the model can be translated into computer code and simulated. To encode the model we used SBML shorthand [37] which can then be converted into the Systems Biology Markup Language (SBML), a computer-readable format for representing biochemical models [38]. The model was imported into the BASIS (Biology of Ageing e-Science Integration and Simulation) system [39,40] and simulations were carried out using a stochastic simulator based on the Gillespie algorithm [41]. The SBML code is provided as supplementary material (see Additional file 1). The model is also in the public repository of BASIS (urn:basis.ncl:model:2215) and will soon be available from the Biomodels database [42,43].

### Elements of the model

It is first necessary to specify all the elements of the model and the interactions between the elements. Using the terminology of SBML, we refer to elements as "species" and interactions as "reactions". The species needed for the model are a pool of native proteins, misfolded proteins, ubiquitin, ubiquitin activating enzyme (E1), ubiquitin conjugating enzyme (E2), ubiquitin ligase (E3), de-ubiquitinating enzymes (DUB), proteasomes, ATP, ADP, AMP, reactive oxygen species (ROS), and complexes to represent binding of elements e.g. ubiquitinated protein. A full list of the species with their names, database terms and their initial amounts is shown in Table 1.

### Interactions between elements of the model

In our previous model of the chaperone system [30], we included a single step for the degradation of misfolded proteins:

MisP+ATP→k6ADP
 MathType@MTEF@5@5@+=feaafiart1ev1aaatCvAUfKttLearuWrP9MDH5MBPbIqV92AaeXatLxBI9gBaebbnrfifHhDYfgasaacH8akY=wiFfYdH8Gipec8Eeeu0xXdbba9frFj0=OqFfea0dXdd9vqai=hGuQ8kuc9pgc9s8qqaq=dirpe0xb9q8qiLsFr0=vr0=vr0dc8meaabaqaciaacaGaaeqabaqabeGadaaakeaacqqGnbqtcqqGPbqAcqqGZbWCcqqGqbaucqGHRaWkcqqGbbqqcqqGubavcqqGqbaudaGdKaWcbaGaem4AaS2aaSbaaWqaaiabiAda2aqabaaaleqakiaawkziaiabbgeabjabbseaejabbcfaqbaa@3D63@

where MisP represents the pool of misfolded protein.

We now replace this single step with a more detailed representation of the ubiquitin-proteasome system.

### Binding of E3 to misfolded protein

The first step in the degradation pathway is that the substrate (in this case MisP) is bound to its specific ubiquitin ligase (E3). The binding of E3 to MisP produces a complex E3_MisP. This reaction is reversible and so we have two reactions as follows:

E3+MisP→k61E3_MisPE3_MisP→k61rE3+MisP
 MathType@MTEF@5@5@+=feaafiart1ev1aaatCvAUfKttLearuWrP9MDH5MBPbIqV92AaeXatLxBI9gBaebbnrfifHhDYfgasaacH8akY=wiFfYdH8Gipec8Eeeu0xXdbba9frFj0=OqFfea0dXdd9vqai=hGuQ8kuc9pgc9s8qqaq=dirpe0xb9q8qiLsFr0=vr0=vr0dc8meaabaqaciaacaGaaeqabaqabeGadaaakqaabeqaaiabbweafjabbodaZiabgUcaRiabb2eanjabbMgaPjabbohaZjabbcfaqnaaoqcaleaacqWGRbWAdaWgaaadbaGaeGOnayJaeGymaedabeaaaSqabOGaayPKHaGaeeyrauKaee4mamJaee4xa8Laeeyta0KaeeyAaKMaee4CamNaeeiuaafabaGaeeyrauKaee4mamJaee4xa8Laeeyta0KaeeyAaKMaee4CamNaeeiuaa1aa4ajaSqaaiabdUgaRnaaBaaameaacqaI2aGncqaIXaqmcqWGYbGCaeqaaaWcbeGccaGLsgcacqqGfbqrcqqGZaWmcqGHRaWkcqqGnbqtcqqGPbqAcqqGZbWCcqqGqbauaaaa@58D0@

We use mass action stochastic kinetics for the rate laws [37]. The binding reaction is a second-order reaction since there are two reactants and is given by *k*_61_<#E3><#MisP>, where # denotes the number of molecules. The disassociation reaction is a first-order reaction and is given by *k*_61*r*_<#E3_MisP>. The values for *k*_61 _and *k*_61*r *_can be estimated from knowledge of the protein half-life and the steady state level of protein in its misfolded state.

### Action of ubiquitin enzymes, E1 and E2 leading to first ubiquitination event

Before a substrate can be ubiquitinated, ubiquitin must be activated by the ubiquitin-activating enzyme (E1). This reaction requires one molecule of ATP. The binding of E1 to ubiquitin (Ub) produces a complex which we denote by E1_Ub:

E1+Ub+ATP→k62E1_Ub+AMP
 MathType@MTEF@5@5@+=feaafiart1ev1aaatCvAUfKttLearuWrP9MDH5MBPbIqV92AaeXatLxBI9gBaebbnrfifHhDYfgasaacH8akY=wiFfYdH8Gipec8Eeeu0xXdbba9frFj0=OqFfea0dXdd9vqai=hGuQ8kuc9pgc9s8qqaq=dirpe0xb9q8qiLsFr0=vr0=vr0dc8meaabaqaciaacaGaaeqabaqabeGadaaakeaacqqGfbqrcqqGXaqmcqGHRaWkcqqGvbqvcqqGIbGycqGHRaWkcqqGbbqqcqqGubavcqqGqbaudaGdKaWcbaGaem4AaS2aaSbaaWqaaiabiAda2iabikdaYaqabaaaleqakiaawkziaiabbweafjabbgdaXiabb+faFjabbwfavjabbkgaIjabgUcaRiabbgeabjabb2eanjabbcfaqbaa@454E@

The activated ubiquitin is then transferred to the ubiquitin-conjugating enzyme (E2) which releases E1 making it available for further ubiquitin-activating reactions:

E2+E1_Ub→k63E2_Ub+E1
 MathType@MTEF@5@5@+=feaafiart1ev1aaatCvAUfKttLearuWrP9MDH5MBPbIqV92AaeXatLxBI9gBaebbnrfifHhDYfgasaacH8akY=wiFfYdH8Gipec8Eeeu0xXdbba9frFj0=OqFfea0dXdd9vqai=hGuQ8kuc9pgc9s8qqaq=dirpe0xb9q8qiLsFr0=vr0=vr0dc8meaabaqaciaacaGaaeqabaqabeGadaaakeaacqqGfbqrcqqGYaGmcqGHRaWkcqqGfbqrcqqGXaqmcqqGFbWxcqqGvbqvcqqGIbGydaGdKaWcbaGaem4AaS2aaSbaaWqaaiabiAda2iabiodaZaqabaaaleqakiaawkziaiabbweafjabbkdaYiabb+faFjabbwfavjabbkgaIjabgUcaRiabbweafjabbgdaXaaa@42FB@

Although it is possible that this reaction is reversible, it seems unlikely that E2 would return the Ub to E1. So we assume that the reverse reaction is negligible and do not include it in the model. Ubiquitin bound to E2 is then transferred to the misfolded protein bound to E3, releasing E2 and E3:

E2_Ub+E3_MisP→k64MisP_Ub+E2+E3
 MathType@MTEF@5@5@+=feaafiart1ev1aaatCvAUfKttLearuWrP9MDH5MBPbIqV92AaeXatLxBI9gBaebbnrfifHhDYfgasaacH8akY=wiFfYdH8Gipec8Eeeu0xXdbba9frFj0=OqFfea0dXdd9vqai=hGuQ8kuc9pgc9s8qqaq=dirpe0xb9q8qiLsFr0=vr0=vr0dc8meaabaqaciaacaGaaeqabaqabeGadaaakeaacqqGfbqrcqqGYaGmcqqGFbWxcqqGvbqvcqqGIbGycqGHRaWkcqqGfbqrcqqGZaWmcqqGFbWxcqqGnbqtcqqGPbqAcqqGZbWCcqqGqbaudaGdKaWcbaGaem4AaS2aaSbaaWqaaiabiAda2iabisda0aqabaaaleqakiaawkziaiabb2eanjabbMgaPjabbohaZjabbcfaqjabb+faFjabbwfavjabbkgaIjabgUcaRiabbweafjabbkdaYiabgUcaRiabbweafjabbodaZaaa@4F48@

### Extending the ubiquitin chain

Further ubiquitin is then attached to the monoubiquitinated protein via the action of E2. Since each ubiquitin molecule must be activated by E1 before being transferred to E2, each step in the formation of polyubiquitin chain uses one molecule of ATP. The extension of the ubiquitin chain is modelled by the following set of reactions:

MisP_Ub+E2_Ub→k65MisP_Ub2+E2MisP_Ub(i)+E2_Ub→k65MisP_Ub(i+1)+E2(i=2,...7)
 MathType@MTEF@5@5@+=feaafiart1ev1aaatCvAUfKttLearuWrP9MDH5MBPbIqV92AaeXatLxBI9gBaebbnrfifHhDYfgasaacH8akY=wiFfYdH8Gipec8Eeeu0xXdbba9frFj0=OqFfea0dXdd9vqai=hGuQ8kuc9pgc9s8qqaq=dirpe0xb9q8qiLsFr0=vr0=vr0dc8meaabaqaciaacaGaaeqabaqabeGadaaakeaafaqaaeGacaaabaGaeeyta0KaeeyAaKMaee4CamNaeeiuaaLaee4xa8LaeeyvauLaeeOyaiMaey4kaSIaeeyrauKaeeOmaiJaee4xa8LaeeyvauLaeeOyai2aa4ajaSqaaiabdUgaRnaaBaaameaacqaI2aGncqaI1aqnaeqaaaWcbeGccaGLsgcacqqGnbqtcqqGPbqAcqqGZbWCcqqGqbaucqqGFbWxcqqGvbqvcqqGIbGycqqGYaGmcqGHRaWkcqqGfbqrcqqGYaGmaeaaaeaacqqGnbqtcqqGPbqAcqqGZbWCcqqGqbaucqqGFbWxcqqGvbqvcqqGIbGycqqGOaakcqqGPbqAcqqGPaqkcqGHRaWkcqqGfbqrcqqGYaGmcqqGFbWxcqqGvbqvcqqGIbGydaGdKaWcbaGaem4AaS2aaSbaaWqaaiabiAda2iabiwda1aqabaaaleqakiaawkziaiabb2eanjabbMgaPjabbohaZjabbcfaqjabb+faFjabbwfavjabbkgaIjabbIcaOiabbMgaPjabgUcaRiabbgdaXiabbMcaPiabgUcaRiabbweafjabbkdaYaqaaiabbIcaOiabbMgaPjabg2da9iabbkdaYiabbYcaSiabb6caUiabb6caUiabb6caUiabbEda3iabbMcaPaaaaaa@7F72@

Each of the above reactions is reversible, with the reversible reactions requiring the activity of a deubiquitinating enzyme (DUB). We assume that the shortening of ubiquitin chains by DUBs is a step-wise process, although we could later modify the model to allow whole chains to be removed if it becomes of interest to model this. The step-wise removal of ubiquitin is modelled by the following reactions:

MisP_Ub(i)+DUB→k66MisP_Ub(i-1)+DUB(i=8,...2)
 MathType@MTEF@5@5@+=feaafiart1ev1aaatCvAUfKttLearuWrP9MDH5MBPbIqV92AaeXatLxBI9gBaebbnrfifHhDYfgasaacH8akY=wiFfYdH8Gipec8Eeeu0xXdbba9frFj0=OqFfea0dXdd9vqai=hGuQ8kuc9pgc9s8qqaq=dirpe0xb9q8qiLsFr0=vr0=vr0dc8meaabaqaciaacaGaaeqabaqabeGadaaakeaafaqabeqacaaabaGaeeyta0KaeeyAaKMaee4CamNaeeiuaaLaee4xa8LaeeyvauLaeeOyaiMaeeikaGIaeeyAaKMaeeykaKIaey4kaSIaeeiraqKaeeyvauLaeeOqai0aa4ajaSqaaiabdUgaRnaaBaaameaacqaI2aGncqaI2aGnaeqaaaWcbeGccaGLsgcacqqGnbqtcqqGPbqAcqqGZbWCcqqGqbaucqqGFbWxcqqGvbqvcqqGIbGycqqGOaakcqqGPbqAcqqGTaqlcqqGXaqmcqqGPaqkcqGHRaWkcqqGebarcqqGvbqvcqqGcbGqaeaacqqGOaakcqqGPbqAcqGH9aqpcqqG4aaocqqGSaalcqqGUaGlcqqGUaGlcqqGUaGlcqqGYaGmcqqGPaqkaaaaaa@5D29@

### Binding to the proteasome and degradation

We assume that ubiquitinated protein first binds to the proteasome and waits for degradation and that it can remain bound so long as at least 4 ubiquitin sub-units are attached. This can be modelled by the following reactions:

MisP_Ub(i)+Proteasome→k67MisP_Ub(i)_Proteasome(i=4,...,8)
 MathType@MTEF@5@5@+=feaafiart1ev1aaatCvAUfKttLearuWrP9MDH5MBPbIqV92AaeXatLxBI9gBaebbnrfifHhDYfgasaacH8akY=wiFfYdH8Gipec8Eeeu0xXdbba9frFj0=OqFfea0dXdd9vqai=hGuQ8kuc9pgc9s8qqaq=dirpe0xb9q8qiLsFr0=vr0=vr0dc8meaabaqaciaacaGaaeqabaqabeGadaaakeaafaqabeqacaaabaGaeeyta0KaeeyAaKMaee4CamNaeeiuaaLaee4xa8LaeeyvauLaeeOyaiMaeeikaGIaeeyAaKMaeeykaKIaey4kaSIaeeiuaaLaeeOCaiNaee4Ba8MaeeiDaqNaeeyzauMaeeyyaeMaee4CamNaee4Ba8MaeeyBa0Maeeyzau2aa4ajaSqaaiabdUgaRnaaBaaameaacqaI2aGncqaI3aWnaeqaaaWcbeGccaGLsgcacqqGnbqtcqqGPbqAcqqGZbWCcqqGqbaucqqGFbWxcqqGvbqvcqqGIbGycqqGOaakcqqGPbqAcqqGPaqkcqqGFbWxcqqGqbaucqqGYbGCcqqGVbWBcqqG0baDcqqGLbqzcqqGHbqycqqGZbWCcqqGVbWBcqqGTbqBcqqGLbqzaeaacqqGOaakcqqGPbqAcqGH9aqpcqqG0aancqqGSaalcqqGUaGlcqqGUaGlcqqGUaGlcqqGSaalcqqG4aaocqqGPaqkaaaaaa@7119@

where MisP_Ub4_Proteasome etc. represents an ubiquitinated protein bound to the proteasome.

Bound ubiquitinated protein can also be de-ubiquitinated in a step-wise process, so that proteins with longer chains stay longer at the proteasome. This is modelled by the following set of reactions:

MisP_Ub(i)_Proteasome+DUB→k68MisP_Ub(i-1)_Proteasome+Ub+DUB(i=8,...,5)
 MathType@MTEF@5@5@+=feaafiart1ev1aaatCvAUfKttLearuWrP9MDH5MBPbIqV92AaeXatLxBI9gBaebbnrfifHhDYfgasaacH8akY=wiFfYdH8Gipec8Eeeu0xXdbba9frFj0=OqFfea0dXdd9vqai=hGuQ8kuc9pgc9s8qqaq=dirpe0xb9q8qiLsFr0=vr0=vr0dc8meaabaqaciaacaGaaeqabaqabeGadaaakeaafaqabeqacaaabaGaeeyta0KaeeyAaKMaee4CamNaeeiuaaLaee4xa8LaeeyvauLaeeOyaiMaeeikaGIaeeyAaKMaeeykaKIaee4xa8LaeeiuaaLaeeOCaiNaee4Ba8MaeeiDaqNaeeyzauMaeeyyaeMaee4CamNaee4Ba8MaeeyBa0MaeeyzauMaey4kaSIaeeiraqKaeeyvauLaeeOqai0aa4ajaSqaaiabdUgaRnaaBaaameaacqaI2aGncqaI4aaoaeqaaaWcbeGccaGLsgcacqqGnbqtcqqGPbqAcqqGZbWCcqqGqbaucqqGFbWxcqqGvbqvcqqGIbGycqqGOaakcqqGPbqAcqqGTaqlcqqGXaqmcqqGPaqkcqqGFbWxcqqGqbaucqqGYbGCcqqGVbWBcqqG0baDcqqGLbqzcqqGHbqycqqGZbWCcqqGVbWBcqqGTbqBcqqGLbqzcqGHRaWkcqqGvbqvcqqGIbGycqGHRaWkcqqGebarcqqGvbqvcqqGcbGqaeaacqqGOaakcqqGPbqAcqGH9aqpcqqG4aaocqqGSaalcqqGUaGlcqqGUaGlcqqGUaGlcqqGSaalcqqG1aqncqqGPaqkaaaaaa@7F02@

When a bound protein has a chain shorter than four, then it dissociates from the proteasome. This is shown in the following reaction:

MisP_Ub4_Proteasome+DUB→k68MisP_Ub3+Proteasome+DUB
 MathType@MTEF@5@5@+=feaafiart1ev1aaatCvAUfKttLearuWrP9MDH5MBPbIqV92AaeXatLxBI9gBaebbnrfifHhDYfgasaacH8akY=wiFfYdH8Gipec8Eeeu0xXdbba9frFj0=OqFfea0dXdd9vqai=hGuQ8kuc9pgc9s8qqaq=dirpe0xb9q8qiLsFr0=vr0=vr0dc8meaabaqaciaacaGaaeqabaqabeGadaaakeaacqqGnbqtcqqGPbqAcqqGZbWCcqqGqbaucqqGFbWxcqqGvbqvcqqGIbGycqqG0aancqqGFbWxcqqGqbaucqqGYbGCcqqGVbWBcqqG0baDcqqGLbqzcqqGHbqycqqGZbWCcqqGVbWBcqqGTbqBcqqGLbqzcqGHRaWkcqqGebarcqqGvbqvcqqGcbGqdaGdKaWcbaGaem4AaS2aaSbaaWqaaiabiAda2iabiIda4aqabaaaleqakiaawkziaiabb2eanjabbMgaPjabbohaZjabbcfaqjabb+faFjabbwfavjabbkgaIjabbodaZiabgUcaRiabbcfaqjabbkhaYjabb+gaVjabbsha0jabbwgaLjabbggaHjabbohaZjabb+gaVjabb2gaTjabbwgaLjabgUcaRiabbseaejabbwfavjabbkeacbaa@6AD6@

Any protein which has a chain of four or more ubiquitin molecules may be degraded by the proteasome in an ATP dependent reaction:

MisP_Ub(i)_Proteasome+ATP→k69iUb+Proteasome+ADP(i=4,...,8)
 MathType@MTEF@5@5@+=feaafiart1ev1aaatCvAUfKttLearuWrP9MDH5MBPbIqV92AaeXatLxBI9gBaebbnrfifHhDYfgasaacH8akY=wiFfYdH8Gipec8Eeeu0xXdbba9frFj0=OqFfea0dXdd9vqai=hGuQ8kuc9pgc9s8qqaq=dirpe0xb9q8qiLsFr0=vr0=vr0dc8meaabaqaciaacaGaaeqabaqabeGadaaakeaafaqabeqacaaabaGaeeyta0KaeeyAaKMaee4CamNaeeiuaaLaee4xa8LaeeyvauLaeeOyaiMaeeikaGIaeeyAaKMaeeykaKIaee4xa8LaeeiuaaLaeeOCaiNaee4Ba8MaeeiDaqNaeeyzauMaeeyyaeMaee4CamNaee4Ba8MaeeyBa0MaeeyzauMaey4kaSIaeeyqaeKaeeivaqLaeeiuaa1aa4ajaSqaaiabdUgaRnaaBaaameaacqaI2aGncqaI5aqoaeqaaaWcbeGccaGLsgcacqqGPbqAcqqGvbqvcqqGIbGycqGHRaWkcqqGqbaucqqGYbGCcqqGVbWBcqqG0baDcqqGLbqzcqqGHbqycqqGZbWCcqqGVbWBcqqGTbqBcqqGLbqzcqGHRaWkcqqGbbqqcqqGebarcqqGqbauaeaacqqGOaakcqqGPbqAcqGH9aqpcqqG0aancqqGSaalcqqGUaGlcqqGUaGlcqqGUaGlcqqGSaalcqqG4aaocqqGPaqkaaaaaa@717C@

### Protein synthesis and refolding

This model can either be combined with the chaperone model [30] or can be explored as a separate module. In order to investigate how the various parameters and species amounts affect the model predictions for the rate of protein turnover, we first carried out simulations on the separate module. In order to do this we added the reactions of protein synthesis, protein misfolding and protein refolding to the model:

→k1NatP,NatP+ROS→k2MisP+ROS,MisP→k3NatP.
 MathType@MTEF@5@5@+=feaafiart1ev1aaatCvAUfKttLearuWrP9MDH5MBPbIqV92AaeXatLxBI9gBaebbnrfifHhDYfgasaacH8akY=wiFfYdH8Gipec8Eeeu0xXdbba9frFj0=OqFfea0dXdd9vqai=hGuQ8kuc9pgc9s8qqaq=dirpe0xb9q8qiLsFr0=vr0=vr0dc8meaabaqaciaacaGaaeqabaqabeGadaaakqaabeqaamaaoqcaleaacqWGRbWAdaWgaaadbaGaeGymaedabeaaaSqabOGaayPKHaGaeeOta4KaeeyyaeMaeeiDaqNaeeiuaaLaeeilaWcabaGaeeOta4KaeeyyaeMaeeiDaqNaeeiuaaLaey4kaSIaeeOuaiLaee4ta8Kaee4uam1aa4ajaSqaaiabdUgaRnaaBaaameaacqaIYaGmaeqaaaWcbeGccaGLsgcacqqGnbqtcqqGPbqAcqqGZbWCcqqGqbaucqGHRaWkcqqGsbGucqqGpbWtcqqGtbWucqqGSaalaeaacqqGnbqtcqqGPbqAcqqGZbWCcqqGqbaudaGdKaWcbaGaem4AaS2aaSbaaWqaaiabiodaZaqabaaaleqakiaawkziaiabb6eaojabbggaHjabbsha0jabbcfaqjabc6caUaaaaa@5D99@

The rate of misfolding depends on the level of reactive oxygen species (ROS) within the cell. We have omitted details of the chaperone activity, where a misfolded protein would bind to Hsp90 before refolding can take place. However, when we combine the models, this simple reaction will be replaced with the more detailed reactions given in Proctor et al., [30]. In this model, we assume that ROS takes a constant value throughout the simulation but we can look at the effect of varying the amount of ROS. It would also be simple to add reactions for the generation and removal of ROS which would then make it possible to examine the effects of increasing ROS over time.

### Protein aggregation

If a misfolded protein is not removed immediately by refolding or degradation, then there is a chance that it will interact with another misfolded protein to form a small aggregate, or it may interact with an existing aggregate to form a larger aggregate. (Here we will not be concerned with the size of aggregates – a more detailed model is in preparation). An aggregate may be sequestered to prevent it interfering with the cellular machinery or it may bind to the proteasome. We include enough detail to be able to examine whether an increase in misfolding (for example by an increase in levels of ROS) leads to an increase in aggregation and inhibition of the proteasome which in turn leads to an even greater level of aggregated protein.

The following set of reactions show how protein aggregation can be simply modelled:

2MisP→k71AggPMisP+AggP→k712AggPAggP→k73SeqAggPAggP+Proteasome→k74AggP_Proteasome
 MathType@MTEF@5@5@+=feaafiart1ev1aaatCvAUfKttLearuWrP9MDH5MBPbIqV92AaeXatLxBI9gBaebbnrfifHhDYfgasaacH8akY=wiFfYdH8Gipec8Eeeu0xXdbba9frFj0=OqFfea0dXdd9vqai=hGuQ8kuc9pgc9s8qqaq=dirpe0xb9q8qiLsFr0=vr0=vr0dc8meaabaqaciaacaGaaeqabaqabeGadaaakqaabeqaaiabbkdaYiabb2eanjabbMgaPjabbohaZjabbcfaqnaaoqcaleaacqWGRbWAdaWgaaadbaGaeG4naCJaeGymaedabeaaaSqabOGaayPKHaGaeeyqaeKaee4zaCMaee4zaCMaeeiuaafabaGaeeyta0KaeeyAaKMaee4CamNaeeiuaaLaey4kaSIaeeyqaeKaee4zaCMaee4zaCMaeeiuaa1aa4ajaSqaaiabdUgaRnaaBaaameaacqaI3aWncqaIXaqmaeqaaaWcbeGccaGLsgcacqqGYaGmcqqGbbqqcqqGNbWzcqqGNbWzcqqGqbauaeaacqqGbbqqcqqGNbWzcqqGNbWzcqqGqbaudaGdKaWcbaGaem4AaS2aaSbaaWqaaiabiEda3iabiodaZaqabaaaleqakiaawkziaiabbofatjabbwgaLjabbghaXjabbgeabjabbEgaNjabbEgaNjabbcfaqbqaaiabbgeabjabbEgaNjabbEgaNjabbcfaqjabgUcaRiabbcfaqjabbkhaYjabb+gaVjabbsha0jabbwgaLjabbggaHjabbohaZjabb+gaVjabb2gaTjabbwgaLnaaoqcaleaacqWGRbWAdaWgaaadbaGaeG4naCJaeGinaqdabeaaaSqabOGaayPKHaGaeeyqaeKaee4zaCMaee4zaCMaeeiuaaLaee4xa8LaeeiuaaLaeeOCaiNaee4Ba8MaeeiDaqNaeeyzauMaeeyyaeMaee4CamNaee4Ba8MaeeyBa0Maeeyzaugaaaa@910B@

where AggP represents the pool of aggregates. The aggregates may be sequestered (SeqAggP) which keeps them out of harms way, or they may bind to the proteasome (AggP_Proteasome) and so inhibit its function. Ubiquitinated misfolded protein may also form aggregates but this will not normally occur unless the proteasome is inhibited. As there are many ways in which misfolded proteins with ubiquitin chains of different lengths can interact, we only list a subset of the reactions:

2MisP_Ub→k72AggPMisP_Ub+AggP→k722AggP⋮MisP_Ub8+AggP→k722AggPMisP+MisP_Ub→k72AggP⋮MisP+MisP_Ub8→k72AggP⋮MisP_Ub7+MisP_Ub8→k72AggP 
 MathType@MTEF@5@5@+=feaafiart1ev1aaatCvAUfKttLearuWrP9MDH5MBPbIqV92AaeXatLxBI9gBaebbnrfifHhDYfgasaacH8akY=wiFfYdH8Gipec8Eeeu0xXdbba9frFj0=OqFfea0dXdd9vqai=hGuQ8kuc9pgc9s8qqaq=dirpe0xb9q8qiLsFr0=vr0=vr0dc8meaabaqaciaacaGaaeqabaqabeGadaaakqaabeqaaiabbkdaYiabb2eanjabbMgaPjabbohaZjabbcfaqjabb+faFjabbwfavjabbkgaInaaoqcaleaacqWGRbWAdaWgaaadbaGaeG4naCJaeGOmaidabeaaaSqabOGaayPKHaGaeeyqaeKaee4zaCMaee4zaCMaeeiuaafabaGaeeyta0KaeeyAaKMaee4CamNaeeiuaaLaee4xa8LaeeyvauLaeeOyaiMaey4kaSIaeeyqaeKaee4zaCMaee4zaCMaeeiuaa1aa4ajaSqaaiabdUgaRnaaBaaameaacqaI3aWncqaIYaGmaeqaaaWcbeGccaGLsgcacqqGYaGmcqqGbbqqcqqGNbWzcqqGNbWzcqqGqbauaeaacqWIUlstaeaacqqGnbqtcqqGPbqAcqqGZbWCcqqGqbaucqqGFbWxcqqGvbqvcqqGIbGycqqG4aaocqGHRaWkcqqGbbqqcqqGNbWzcqqGNbWzcqqGqbaudaGdKaWcbaGaem4AaS2aaSbaaWqaaiabiEda3iabikdaYaqabaaaleqakiaawkziaiabbkdaYiabbgeabjabbEgaNjabbEgaNjabbcfaqbqaaiabb2eanjabbMgaPjabbohaZjabbcfaqjabgUcaRiabb2eanjabbMgaPjabbohaZjabbcfaqjabb+faFjabbwfavjabbkgaInaaoqcaleaacqWGRbWAdaWgaaadbaGaeG4naCJaeGOmaidabeaaaSqabOGaayPKHaGaeeyqaeKaee4zaCMaee4zaCMaeeiuaafabaGaeSO7I0eabaGaeeyta0KaeeyAaKMaee4CamNaeeiuaaLaey4kaSIaeeyta0KaeeyAaKMaee4CamNaeeiuaaLaee4xa8LaeeyvauLaeeOyaiMaeeioaGZaa4ajaSqaaiabdUgaRnaaBaaameaacqaI3aWncqaIYaGmaeqaaaWcbeGccaGLsgcacqqGbbqqcqqGNbWzcqqGNbWzcqqGqbauaeaacqWIUlstaeaacqqGnbqtcqqGPbqAcqqGZbWCcqqGqbaucqqGFbWxcqqGvbqvcqqGIbGycqqG3aWncqGHRaWkcqqGnbqtcqqGPbqAcqqGZbWCcqqGqbaucqqGFbWxcqqGvbqvcqqGIbGycqqG4aaodaGdKaWcbaGaem4AaS2aaSbaaWqaaiabiEda3iabikdaYaqabaaaleqakiaawkziaiabbgeabjabbEgaNjabbEgaNjabbcfaqjabbccaGaaaaa@CB93@

### Setting the initial amounts of the species and parameter values

Before the model can be simulated, it is necessary to specify the initial amounts of each species and the parameter values. There is experimental data on levels of ubiquitin and proteasomes in human cells. For example, Haas et al. [44] measured levels of monomeric ubiquitin and conjugated ubiquitin in IMR-90 cells giving values of 71.5 pmol/10^6 ^cells and 61.1 pmol/10^6 ^cells respectively. From this we can calculate that there are approximately 10^7 ^ubiquitin molecules per cell (estimated at between 1 and 10% of total protein molecules). Since we are only modelling part of the total cellular protein, we have scaled down the level of ubiquitin to 500 molecules per cell. The total level of proteasomes per cell has been estimated to be 8 × 10^5 ^per cell in L929 cells [34] which gives a ratio of about one proteasome to every 10 ubiquitin molecules. Since ubiquitin has many other functions apart from the ubiquitin-proteasome pathway, the ratio of proteasomes to ubiquitin must be less than 1:10 and so we assumed a ratio of 1:5.

The level of E1 has been measured in IMR90 cells by western analysis of cell lysate compared to recombinant protein (Tsirigotis and Gray, unpublished data) and is estimated to be 1 million molecules per cell. This is one order less than the level of ubiquitin and so we chose an initial amount of 100 for E1. We do not have data for E2 or E3 enzymes but for the sake of building the model assume that for specific substrates these would be present at roughly the same abundance as E1. We do not have any experimental data for the abundance of DUBs within cells but assume that these are less abundant than ubiquitin and set the initial amount to 200.

The level of native protein, NatP, was set at 500 and at this level the ratio of native protein to ubiquitin and proteasomes enabled efficient degradation to take place. If we increased the level of NatP to 5000, then it was also necessary to increase the pool of ubiquitin and proteasomes to prevent depletion of monomeric ubiquitin under normal conditions. The levels of ATP, ADP and AMP were set at a fairly arbitrary level of 10000, 1000 and 1000 respectively and the levels were kept constant by imposing a boundary condition to these species. We chose to do this instead of allowing the levels to fluctuate as cells possess mechanisms to maintain ATP levels at fairly constant levels and it is unlikely that the ubiquitin-proteasome system would cause major changes to levels of these molecules. ROS levels were set at an arbitrary value of 10 and the level remained constant as there are no mechanisms in this model that would cause ROS levels to change. The initial amounts of all the species are shown in Table 1.

The default values for the model parameters are shown in Tables 2 and 3. Where possible values were chosen to reflect experimental measurements. For example, the parameters for protein synthesis and misfolding can be calculated from knowledge of the protein half-life and the steady state levels of misfolded protein. Other parameter values such as those for the ubiquitin pathway are currently unknown but experimental work in the lab of DG is being carried out to quantify some of the reactions rates. Before this data becomes available, we try to find a set of parameters that will give output corresponding to the expected steady state of an unstressed cell e.g. no accumulation of misfolded protein and only small fluctuations in levels of native protein, ATP, ADP and ROS. To do this we initially estimate the unknown parameters and then run a simulation and check the model output. The parameters shown in Tables 2 and 3 corresponded to a steady state as shown in Figure 2b. We then investigated which parameters affected the half-life of the protein substrate by setting the rate of protein synthesis to zero and altering each of the other parameters in turn. We also examined the effects of inhibiting the proteasome by setting the parameter for degradation to zero and the effects of shutting down E1 activity. For example, with our original choice of parameters our model predicted that if E1 activity is shut down, then there is an almost immediate conversion of ubiquitin conjugates to monomeric ubiquitin. However, experimental data shows that this conversion takes about two hours (Fig 5a). Therefore, we adjusted the parameters to reduce the rates of the reactions involved in ubiquitination, de-ubiquitination and degradation until the model output fitted the experimental data.

We now describe each of the parameters in turn and give an interpretation of their values in terms of reaction kinetics. The parameters *k*_1_, *k*_2 _and *k*_3 _were set so that the half-life of the pool of protein equals about 10 hours, and under normal conditions, there is only a very low proportion of misfolded protein [45]. The parameter *k*_61 _for E3/MisP binding is set so that the average time for one E3 to bind to MisP is about 10 minutes. The parameter *k*_61*r *_is set so that the reverse reaction (dissociation of E3 from MisP) happens once every 100 minutes. So the forward reaction is 10 times stronger than the reverse reaction. The parameter *k*_62 _for E1/Ub binding is set so that about 40 ubiquitin molecules are activated every minute and the parameter *k*_63 _for E2/Ub binding is set to give about 6 reactions per minute. The parameter *k*_64 _for monoubiquitination is set so that once MisP is bound by E3 it takes about 10 seconds to receive the first Ub molecule. The parameter *k*_65 _for polyubiquitination is set so that it took about one second for each additional Ub molecule to be added to the chain. So each polyubiquitination step is about 10 times faster than the first ubiquitination reaction. The parameter *k*_66 _for the de-ubiquitination reactions is set so that it takes about 8 minutes for each reaction. It is necessary that this reaction is very slow compared to the ubiquitination reactions so that the model output fits the experimental data when E1 activity is shut down (see section 4.4). The parameter *k*_67 _for the binding of ubiquitinated proteins (with chains of 4 or more ubiquitin molecules) to the proteasome is set so that under normal conditions when there are about 200 ubiquitin conjugates present in the cell, on average about 5 reactions per second are occurring. The parameter *k*_68 _for the de-ubiquitination of proteasome-bound ubiquitinated proteins is set to be equal to *k*_66_. We assume that this reaction is slow so that proteins with long chains are highly likely to reside long enough at the proteasome for degradation to take place, whereas proteins with short chains may be shortened to below the required threshold and would then dissociate from the proteasome. The parameter *k*_69 _for proteasome activity is set so that it takes about 15 seconds to degrade each proteasome-bound ubiquitinated protein. We initially set *k*_71 _and *k*_72 _to be equal to 10^-7 ^as in the model of Proctor et al. [30]. However, with these values, aggregates sometimes formed even under normal conditions (data not shown). Therefore we lowered the values until we obtained results in which the appearance of aggregates was very unlikely under normal conditions. This was satisfied with *k*_71 _and *k*_72 _set equal to 10^-8^, The parameter *k*_73 _for sequestering aggregates is set so that if there is a low level of aggregates in the cell (1–10), then on average there are 0.005 reactions per second, so each reaction takes about 3 minutes for each reaction.

The parameter *k*_74 _is set so that there is an equal probability of an aggregated protein being sequestered or binding to the proteasome when all the proteasomes are available for binding.

### Adapting the model to mimic different experimental procedures

#### Proteasome inhibition

We carried out an *in silico *experiment of inhibiting the proteasome by setting the parameter *k*_69 _= 0. We also varied each of the other parameters in turn to see which parameters affect the aggregation kinetics.

#### Blocking protein synthesis

By setting the parameter for protein synthesis, *k*_1_, to zero we can use our model to check the half-life of the protein pool. We also varied each of the other parameters in turn to see which parameters affect the half-life. For each parameter we increased and decreased its value ten-fold, re-ran the simulations and plotted the model output.

#### Shutting down E1 activity

It is very straight forward to mimic this experiment with our *in silico *model by adding an event to the SBML code so that at a certain time-point the parameter for the E1/Ub binding reaction is set to zero. This event is equivalent to putting the cells at the non-permissive temperature in the wet lab experiment. We chose 30 minutes as the time-point at which this event occurred to allow time for ubiquitin conjugates to form. The details of the event are given in Table 4.

### Statistical analysis of repeat runs

Statistical analysis of repeated simulations was carried using the R programming language.

## Authors' contributions

CJP constructed and coded the models, ran simulations, plotted results and drafted the manuscript. MT designed and performed laboratory experiments and wrote sections of the manuscript. DAG designed the experiments, advised on the model construction and contributed sections of the manuscript. All authors read and approved the final manuscript.

## Supplementary Material

Additional File 1 Proctor2007_UPSProteinDegradation.xml. SBML code for the ubiquitin-proteasome model Click here for file

## Figures and Tables

**Table 1:** List of species with database terms and initial conditions

Species description	Species Name	Database term	Initial Amount
Native protein	NatP	N/A	500
Misfolded protein	MisP	N/A	0
Ubiquitin	Ub	IPR000626	500
Ubiquitin activating enzyme	E1	IPR000011	100
Ubiquitin conjugating enzyme	E2	IPR000608	100
Ubiquitin ligase (HECT)	E3	IPR000569	100
De-ubiquitinating enzyme	DUB	IPR001394	200
Proteasome	Proteasome	GO:0000502	100
Reactive oxygen species	ROS	CHEBI:26523	10
Adenosine triphosphate	ATP	CHEBI:15422	10000
Adenosine diphosphate	ADP	CHEBI:16761	1000
Adenosine monophosphate	AMP	CHEBI:22254	1000
Misfolded protein bound by E3	E3_MisP	IPR000569	0
E1 bound by Ub	E1_Ub	IPR000011	0
E2 bound by Ub	E2_Ub	IPR000608	0
Monoubiquitinated misfolded protein	MisP_Ub	IPR000626	0
Polyubiquitinated misfolded protein	MisP_Ub2, ... MisP_Ub8	IPR000626	0
Polyubiquitinated protein bound to proteasome	MisP_Ub4_Proteasome ... MisP_Ub8_Proteasome	IPR000626 GO:0000502	0
Aggregated protein	AggP	N/A	0
Sequestered aggregated protein	SeqAggP	N/A	0
Aggregated protein bound to proteasome	AggP_Proteasome	GO:0000502	0
Dummy species to track chain length of substrate at degradation	degUb4, ..., degUb8	N/A	0

IPR: InterPro [46]

GO: Gene ontology [47]

CHEBI: Chemical Entities of Biological Interest database [48]

**Table 2:** List of reactions and parameter values for the ubiquitin-proteasome system

Reaction	Kinetic rate law	Reactants	Products	Parameter name	Value^a^
Protein synthesis	*k*_1_		NatP	*k*_1_	0.01 mol s^-1^
Misfolding	*k*_2_<#NatP><#ROS>	NatP, ROS	MisP, ROS	*k*_2_	2.0E-6 mol^-1 ^s^-1^
Refolding	*k*_3 _<#MisP>	MisP	NatP	*k*_3_	4.0E-6 s^-1^
MisP/E3 binding	*k*_61 _<#E3><#MisP>	E3, MisP	E3_MisP	*k*_61_	1.7E-5 mol^-1 ^s^-1^
MisPE3 release	*k*_61*r *_<#E3_MisP>	E3_MisP	E3, MisP	*k*_61*r*_	2.0E-4 s^-1^
E1/Ub binding	*k*_62 _<#E1><#Ub><#ATP>/(5000+<#ATP>)	E1, Ub, ATP	E1_Ub, AMP	*k*_62_	2.0E-4 mol^-1 ^s^-1^
E2/Ub binding	*k*_63 _<#E2><#E1_Ub>	E2, E1_Ub	E1, E2_Ub	*k*_63_	1.0E-3 mol^-1 ^s^-1^
Monoubiquitination	*k*_64 _<#E2_Ub><#E3_MisP>	E2_Ub, E3_MisP	E2, E3, MisP_Ub	*k*_64_	1.0E-3 mol^-1 ^s^-1^
Polyubiquitination1	*k*_65 _<#E2_Ub><#MisP_Ub>	E2_Ub, MisP_Ub	MisP_Ub2	*k*_65_	1.0E-2 mol^-1 ^s^-1^
Polyubiquitination2	*k*_65 _<#E2_Ub><#MisP_Ub2>	E2_Ub, MisP_Ub2	MisP_Ub3	*k*_65_	1.0E-2 mol^-1 ^s^-1^
⋮	⋮	⋮	⋮	⋮	⋮
Polyubiquitination7	*k*_65 _<#E2_Ub><#MisP_Ub8>	E2_Ub, MisP_Ub7	MisP_Ub8	*k*_65_	1.0E-2 mol^-1 ^s^-1^
De-ubiquitination1	*k*_66 _<#DUB><#MisP_Ub8>	DUB, MisP_Ub8	DUB, MisP_Ub7, Ub	*k*_66_	1.0E-5 mol^-1 ^s^-1^
⋮	⋮	⋮	⋮	⋮	⋮
De-ubiquitination8	*k*_66 _<#DUB><#MisP_Ub>	DUB, MisP_Ub	DUB, MisP, Ub	*k*_66_	1.0E-5 mol^-1 ^s^-1^
Proteasome binding1	*k*_67 _<#MisP_Ub4><#Proteasome>	MisP_Ub4, Proteasome	MisP_Ub4_ Proteasome	*k*_67_	1.0E-5 mol^-1 ^s^-1^
⋮	⋮	⋮	⋮	⋮	⋮
Proteasome binding5	*k*_67 _<#MisP_Ub8><#Proteasome>	MisP_Ub8, Proteasome	MisP_Ub8_ Proteasome	*k*_67_	1.0E-5 mol^-1 ^s^-1^
De-ubiquitinationBoundMisP1	*k*_68 _<#DUB><#MisP_Ub4_Proteasome>	DUB, MisP_Ub4_Proteasome	DUB, MisP_Ub3, Ub, Proteasome	*k*_68_	1.0E-5 mol^-1 ^s^-1^
De-ubiquitinationBoundMisP2	*k*_68 _<#DUB><#MisP_Ub5_Proteasome>	DUB, MisP_Ub5_Proteasome	DUB, Ub, MisP_Ub4_Proteasome	*k*_68_	1.0E-5 mol^-1 ^s^-1^
⋮	⋮	⋮	⋮	⋮	⋮
De-ubiquitinationBoundMisP5	*k*_68 _<#DUB><#MisP_Ub8_Proteasome>	DUB, MisP_Ub8_Proteasome	DUB, Ub, MisP_Ub7_Proteasome	*k*_68_	1.0E-5 mol^-1 ^s^-1^
ProteasomeActivity1	*k*_69 _<#MisP_Ub4_Proteasome><#ATP>/(5000+<#ATP>)	ATP, MisP_Ub4_ Proteasome	ADP, Proteasome, 4Ub,	*k*_69_	1.0E-3 s^-1^
⋮	⋮	⋮	⋮	⋮	⋮
ProteasomeActivity 5	*k*_69 _<#MisP_Ub8_Proteasome><#ATP>/(5000+<#ATP>)	ATP, MisP_Ub8_ Proteasome	ADP, Proteasome, 8Ub,	*k*_69_	1.0E-3 s^-1^

^a^mol, number of molecules

**Table 3:** List of reactions and parameter values for the aggregation process

Reaction	Kinetic rate law	Reactants	Products	Parameter name	Value^a^
Aggregation1	*k*_71_<#MisP><#MisP-1>/2.0	2MisP	AggP	*k*_71_	1.0E-8 mol^-1 ^s^-1^
Aggregation2	*k*_71_<#MisP><#AggP>	MisP, AggP	2AggP	*k*_71_	1.0E-8 mol^-1 ^s^-1^
Aggregation3	*k*_72_<#MisP_Ub><#MisP_Ub-1>/2.0	2MisP_Ub	AggP	*k*_72_	1.0E-8 mol^-1 ^s^-1^
⋮	⋮	⋮	⋮	⋮	⋮
Aggregation10	*k*_72_<#MisP_Ub8><#MisP_Ub8-1>/2.0	2MisP_Ub8	AggP	*k*_72_	1.0E-8 mol^-1 ^s^-1^
Aggregation11	*k*_72_<#MisP><#MisP_Ub>	MisP, MisP_Ub	AggP	*k*_72_	1.0E-8 mol^-1 ^s^-1^
⋮	⋮	⋮	⋮	⋮	⋮
Aggregation54	*k*_72_<#MisP_Ub7><#MisP_Ub8>	MisP_Ub7, MisP_Ub8	AggP	*k*_72_	1.0E-8 mol^-1 ^s^-1^
SequesteringOfAggregates	*k*_73_<#AggP>	AggP	SeqAggP	*k*_73_	1.0E-3 s^-1^
ProteasomeInhibition	*k*_74_<#AggP>	AggP, Proteasome	AggP_Proteasome	*k*_74_	1.0E-5 s^-1^

^a^mol, number of molecules

**Table 4:** Event structure for shutting down E1 activity in the computer model

event	trigger	event assignment	reactions affected
Increase temperature	t >= 1800	*k*_62 _= 0	E1/Ub binding
